# Recycling of Nonwoven Polyethylene Terephthalate Textile into Thermal and Acoustic Insulation for More Sustainable Buildings

**DOI:** 10.3390/polym13183090

**Published:** 2021-09-14

**Authors:** David Antolinc, Kristina Eleršič Filipič

**Affiliations:** 1 Faculty of Civil and Geodetic Engineering, University of Ljubljana, Jamova 2, SI-1000 Ljubljana, Slovenia; 2 FILC/Freudenberg, Trata 48, SI-4220 Škofja Loka, Slovenia; kristina.elersic@guest.arnes.si; 3 Molecular Biology Laboratory, BIA Separations CRO, Labena Ltd., SI-1000 Ljubljana, Slovenia

**Keywords:** textile waste, thermal insulation, sound absorber, sustainability, circular economy, recycling

## Abstract

The construction and building sector is responsible for a large share of energy and material used during the life cycle of a building. It is therefore crucial to apply a circular economy model within the process wherever possible to minimize the impact on the environment. In this paper, the possibility of producing thermal and acoustic boards from industrial nonwoven waste textile is studied and presented. The nonwoven polyester textile obtained directly from the production line in the form of strips and bales was first shredded into smaller fractions and then in the form of pile compressed with a hot press to form compact thermal insulation boards. The first set of specimens was prepared only from waste polyester nonwoven textile, whereas the second set was treated with sodium silicate in order to check the material’s reaction to fire performance. The experimental work was conducted to define the acoustic properties, reaction to fire behavior and thermal conductivity of the produced specimens. The obtained results show that the thermal conductivity coefficient of specimens without added water glass dissolution is near to the values of conventional materials used as thermal insulation in buildings. The reaction to fire testing proved that the addition of water glass actually propagates the progressive flame over the entire product. It can be concluded that the presented thermal insulation can be used as an adequate and sustainable solution for building construction purposes.

## 1. Introduction

One of the main challenges and problems of modern society is the ever growing volume of waste generated at all levels and domains of human activity. Another problem is also the increased use of energy resources and the accompanying release of greenhouse gasses such as CO_2_ among others into the air. The improper use of natural resources, secondary raw materials and energy along with the increasing world population is reflected in severe climate changes, loss of biodiversity, land and water pollution [1,2]. The increased awareness about environmental problems addressed over the past decades has unveiled the need for new sustainable solutions to provide opportunities for the next generation to meet their needs in order to achieve their economic, social and environmental objectives. These new sustainable solutions can be achieved by implementing eco-innovations, so-called green innovations or environmental innovations, at the level of companies and countries with considerable environmental impacts [1].

It is important to note that regulations, market pull, and—less frequently but also very importantly—company size, technology push, competition, management and cost savings are the main eco-innovation triggers studied by Hojnik et al. [3]. It is therefore important to have a clear and complete policy and strategy to tackle environmental problems, such as the European Green Deal, which has been developed and published in more documents by the European Commission [2,4,5,6] in recent years. This is a basis for mobilizing industry for a clean and circular economy that supports the eco-innovation with the primary focus on the sectors that use most resources and have a high potential for establishing circular economy [2]. The construction and building sector has a great potential to increase sustainability as it uses large amounts of materials and consumes approximately 40% of all produced energy for the operation of building stock. It is therefore essential to improve the energy efficiency of old buildings in order to meet the ambitious plans to minimize the negative impact on the environment. The installation of sufficient thermal insulation on the building envelope and upgrade to the use of renewable energy sources are the main measures to achieve adequate energy efficiency according to the current standards and to reach the goal of climate neutrality by 2050 [2].

Additionally, a shift from the linear to circular economy plays an important role in achieving this goal. In order to remain in line with the European Green Deal and to reduce the intensive use of raw materials in construction and the building industry, it is crucial to apply circular economy model to ensure that the largest possible amount of secondary raw materials is used for the construction of new and renovating of old buildings [1,2,7,8].

Polyester or specifically polyethylene terephthalate (PET) accounts for a large part (18%) of the global polymer production and around 70% of all polyester produced is used for fiber applications. In view of the urgent need to find the solution for high volumes of textile waste, the main goal of the present study was to demonstrate the possibility of using industrial waste nonwoven textile made of pure PET fibers as a secondary raw material for the production of thermal insulation boards suitable for applications in buildings. This concept covers the above discussed circular economy principle and at the same time enables the sustainability of buildings by saving energy within the building envelope and thus minimizing the environmental impact. In collaboration with the industrial partners FILC/Freudenberg, who is a producer of technical nonwoven textile, and waste management company CEP d.o.o. from Slovenia, thermal and sound insulation boards made of nonwoven waste textile were developed and tested. Every year the production line generates a substantial amount, between 1500 and 2000 t, of nonwoven polyester waste textile as a result of textile formatting at the end of production process or as probe textile sheets with insufficient quality during set up of the production lines, which is then rolled to bales and finally discarded. Currently, the majority of the generated waste nonwoven textile from the production line is incinerated for energy recovery, which is a source of CO_2_ release and an additional cost for the company. The waste nonwoven textile directly from the production line is made of high-quality PET fibers without external dirt and is as such an ideal secondary raw material that can be used to manufacture profitable products.

This is in contrast to apparel waste textiles which are usually composed of two or more materials, such as cotton, polyester, wool, linen, silk and acrylic, where more effort is needed to recycle these materials and use them as secondary raw materials [9]. However, several authors have presented the attempts to produce thermal and noise insulation made of waste apparel textile [9,10,11,12,13,14,15,16,17,18,19,20,21,22]. There are different mechanical and chemical recycling processing technologies available for waste textiles [23]. In direct mechanical methods, the waste textile is first cut into smaller fractions without converting fabrics back into fibers. These smaller fractions can be directly blown into the air gap of an external double masonry wall system as an effective thermal insulation [9] or it has a potential as an insulation material of light timber frame walls and roofs. Trajković et al. [13] have produced and investigated the thermal insulation blankets made of shredded nonwoven polyester apparel textile encased with the 100% polypropylene nonwoven fabric which was stabilized by stitching. To produce thermal insulation materials from waste textile, a nonwoven processing technique requires more processing energy input as the waste fabric must be decomposed down to separate fiber or polymer that is recycled from textile waste. Loose recycled fibers are then used within the standard methods of producing nonwoven textiles [15,16,24]. As tested by several researchers [11,25,26], insulation materials can also be produced as composites by applying heat and pressure [24]. They all have in common that they used mixed waste textile such as nylon and polyurethane [26] or polyester and polyurethane [11] and various other materials and combinations [20,24,25] with or without nonwoven fabric encasing. Another more advanced option for recycling of polymeric waste plastic is chemical method applying the depolymerization with technologies such as hydrolysis, glycolysis, methanolysis, ammonolysis and aminolysis. For the chemical recycling of polyester PET material, including the polyester nonwoven textile, the depolymerization by the glycolysis of diethylene glycol can be effectively used [27,28]. This is a process where the glycolized PET oligomers are first reacted with glycerol to produce polyols, which can be further used to produce polyurethane foam via the reaction between produced polyols and polymeric methylene-4,4′-diphenyl diisocyanate [27]. The final product is a high quality thermal insulation (polyurethane foam) produced from the waste PET fibers or textile. One of the options for textile waste application is also to use it as a filler or reinforcement of organic or inorganic matrices, as described by Patti et al. [29] and Tedesco et al. [30], to improve mechanical characteristics of the produced composites. A successful attempt to use shredded polyester waste textile as aerating agent in the production of aerated clay bricks has also been studied by Tedesco et al. [30]. This shows that there is great interest in finding a solution for waste textile, as it is one of the main sources of waste with 5.8 million tons discarded by consumers each year in Europe alone. Roughly 75% of this waste is incinerated or landfilled and the remaining small proportion of 25% is recycled [9,31].

When processing the nonwoven polyester waste textile, the problem was faced while shredding it into smaller fractions due to the large forces needed to tear it, which can result in temperature rise and consequently melting of the material. This problem was also previously addressed by Trajković et al. [13], who suggested that a two-stage shredder with a special knife must be used to avoid this problem. The fractured waste textile was then further processed with a hot press to obtain compact novel semi rigid thermal insulation boards. There are also some options to significantly improve the thermal performance of raw polyester fibers such as impregnation of fibers with silica aerogel particles, such as presented by Lucci et al. [32,33]. There is also great potential to improve the thermal performance of the shredded waste nonwoven polyester textile in the future research. Based on the conclusion of previous authors [34,35,36] that the addition of water glass could improve the fire resistance of the end product, half of the specimens were treated with a water glass to check the potential beneficial effect on our products. In the following Sections, the methods to identify the material thermal conductivity coefficient, acoustic and fire properties are presented together with the results obtained on the produced thermal insulation boards.

## 2. Materials and Methods

### 2.1. Nonwoven Polyester Waste Textile

**Figure 1:**
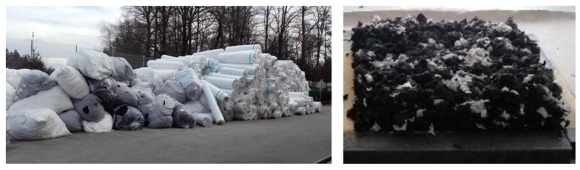
The discarded nonwoven polyester textile at the company’s waste storage (**left**) and shredded waste polyester nonwoven textile prepared for further processing and manufacturing (**right**).

Polyester fibers are widely used for the production of nonwoven industrial textile in the form of sheets or continuous fabric (layer, strip), which is then rolled to the bales at the end of the production process. Short polyester fibers are first spread out in the form of a sheet or web and then bound together with one of the three options. The most commonly used is the mechanical bonding of fibers, whereby they are interlocked with serrated needles. The other two options are binding the fibers with adhesive or thermally with an increase in temperature to melt the binder, where the binder is the additional polymer added to the fibers and binds the fibers together after melting at elevated temperature. After production and prior to rolling to a bale, the fabric is usually formatted to the standard desired dimension (width), which is also the source of the relatively large amount of nonwoven textile waste at the production line. Another source of waste at the production line is related to the change of parameters such as density of the nonwoven textile where usually the initial product does not meet the required parameters and quality. Therefore, this nonwoven textile, which is produced during the setup process, is directly rolled to a bale and discarded. Both sources of nonwoven textile waste from the industry are clean, free of organic substances and relatively homogenous in terms of material, which makes them suitable for direct recycling into a high-quality product. For the purpose of the present study the discarded waste polyester nonwoven textile was provided by the industrial partner and producer of technical nonwoven textile FILC/Freudenberg. Rolls and bales of discarded waste polyester nonwoven textile were taken randomly from the company’s waste storage shown in the left Figure 1. To provide the representative sample of waste polyester textile, approximately 3 tons of material were processed in a two stage shredder to obtain the waste material fractions with the size from 1.5 to 3 cm, as shown in the right Figure 1.

### 2.2. Water Glass

A sodium silicate or water glass is part of the soluble silicates family and is one of the most versatile inorganic chemicals available [36]. Water glass is produced by melting a mixture of caustic soda (NaOH) and quartz sand (SiO_2_) with the end product of sodium metasilicate (Na_2_SiO_3_). Water glass is usually in the form of transparent solid, white powder or soluble in water as viscous liquid with a 25 to 28 wt % SiO_2_ and 6 to 18 wt % Na_2_O. Water glass is widely used in industry as fireproofing and adhesive agent in building materials. For this reason, commercially available soluble sodium metasilicate (Samson Kamnik, Slovenia) in form of viscous liquid was applied with a watering sprayer on the waste polyester flakes to test the fire behavior of the produced thermal insulation.

### 2.3. Method and Process of Thermal Insulation Production

The basic component of the produced thermal insulation slab specimens in this study was shredded nonwoven waste polyester textile obtained from the company FILC/Freudenberg. The nonwoven waste textile was first shredded with a two-stage shredder into the particle size between 1.5 and 3 cm, which were further used to produce two types of insulation slabs. In the first type of slab (designation B1-B8) only shredded nonwoven textile was used without any other substance and in the second type (designation WG1-WG8) water glass was added to the surface of the shredded nonwoven textile particles. The designed dimensions of specimens were 30 cm × 30 cm × 3 cm and 30 cm × 30 cm × 5 cm for the specimens B1-4, WG1-4 and B5-8, WG5-8 respectively.

When preparing the specimens, the main control parameter was density, which was controlled with the mass of shredded nonwoven textile used for the production of thermal insulation slab. The masses of 300, 400, 500 and 700 g were used as the initial amount of shredded nonwoven textile to produce the thermal insulation slabs of different thicknesses and types. The composition details for each produced specimen are summarized in Table 1.

**Figure 2:**
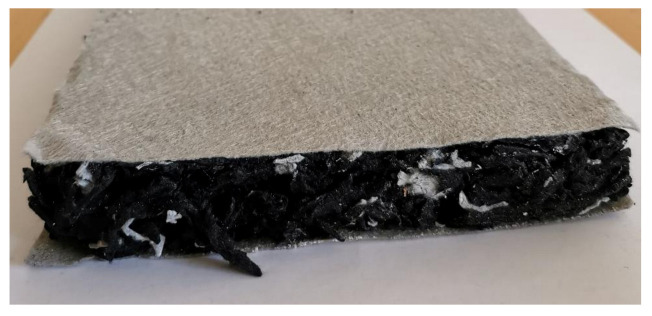
Thermal insulation slab made of shredded compressed waste polyester nonwoven textile.

In the first step of the insulation slab production, the intact nonwoven textile was placed on the table. In the next step, a formwork made of steel profile with the designed final insulation thickness was placed over the intact nonwoven textile. Then the prepared mixture of shredded nonwoven textile with correspondingly defined mass was added into the prepared formwork. The specimen was finally covered by the intact nonwoven textile sheet which provided, after thermal treatment, the reinforcement and smooth top and bottom outer surfaces of the end product. Figure 2 shows the example of the final product.

**Figure 3:**
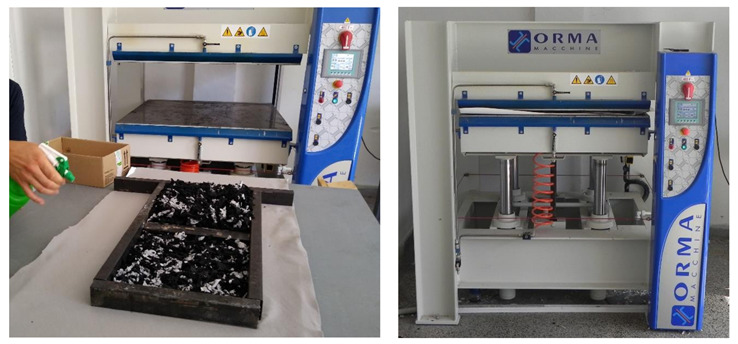
Applying of water glass on the shredded waste nonwoven textile (**left**) and pressing of the prepared material in the preheated hydraulic press (**right**).

**Figure 4:**
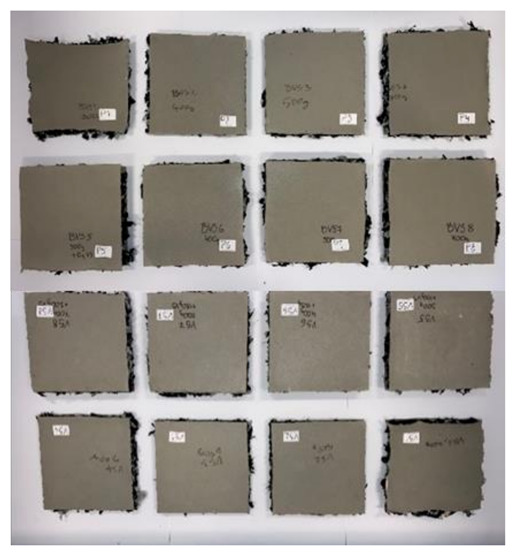
Overview of the produced thermal insulation slab specimens.

The specimens prepared in this way were then transported to the preheated press, which is shown in Figure 3. The compression surfaces of the press were preheated to 240 °C and then compressed down to the steel profile spacer (formwork) which provided us with the desired thickness of the produced thermal insulation slabs. The material was compressed at this temperature for 15 min. The specimens were then taken out of the press and were for further 15 min left lightly compressed while cooling down before they were further handled. The cooling time is needed for the fibers of nonwoven textile to bond together during the solidifying process of the polyester material. For specimens WG1 to WG8 the water glass was applied first to the bottom nonwoven textile sheet and then to each layer of 100 g of added shredded nonwoven waste textile. The 25 g of water glass for each new layer of nonwoven textile was applied with the watering sprayer as it is shown in the left in Figure 3. The end product was a very compact semi rigid insulation slab with a thickness of 3 or 5 cm encased with the polyester nonwoven textile as shown in Figure 2. Overview picture of all 16 produced thermal insulation slabs is shown in Figure 4.

### 2.4. Experimental Methods

In order to evaluate the adequacy of the thermal insulation slabs produced from shredded waste nonwoven textile for the purposes of thermal insulation in buildings, thermal conductivity and reaction to the fire test were conducted. Additionally, the acoustic property test was conducted to check the possibility of using the produced thermal insulation slabs as sound absorbers.

#### 2.4.1. Thermal Conductivity Test

The main objective of the present research was to identify the possibilities of using the produced insulation boards as thermal insulation for buildings. Therefore, the thermal conductivity test was conducted to obtain the thermal conductivity coefficient *λ*.

The thermal conductivity coefficient *λ* of the produced insulation boards with the dimensions of 30 cm × 30 cm and with the thickness of 3 or 5 cm, was conducted with thermal conductivity test using the method of a heat flow meter according to the standard EN 12667:2002 [37]. The heat flow meter apparatus with a single-specimen symmetrical configuration was used, which means that the specimens were placed between the two heat flow meters (AHLBORN, Almemo type 118)(AHLBORN, Holzkirchen, Germany) with a shape of 120 mm × 120 mm rectangular plate, which can also measure the contact temperature of the specimen surface. To establish a constant steady heat flow through the specimen, a thermo-electrical aluminum heating plate was placed on one side and the heat sink on the opposite side of the specimen. On the hot side of the specimen, the temperature was set to 35 °C and on the cold side to 25 °C to achieve the temperature difference of 10 °C, which ensured the steady heat flow through the specimen.

#### 2.4.2. Reaction to Fire Test

**Figure 5:**
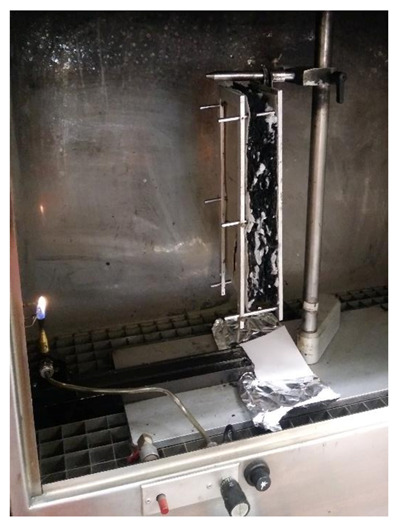
Reaction to fire test set-up for thermal insulation specimens according to the standard ISO 11925-2:2020 [38].

In order to define the burning behavior of the produced insulation boards, the reaction to fire test was conducted according to the standard EN ISO 11925-2 [38]. With this standard testing procedure, the ignitability of building products directly subjected to the impingement of flame is tested. Specimens of a rectangular shape with the dimensions of 250/90 mm were prepared as required by the standard. Specimens were further suspended vertically and directly impinged with a small flame at an angle of 45° to the specimen for 15 s at the bottom edge, as shown in Figure 5. The behavior of the specimen is observed during the exposure to the flame and after removal from the specimen for 20 s.

To classify the specimen into class E, the potential burning of the specimen should extinguish within 20 s after the flame removal and the flame spread should not exceed 150 mm limit marked along with the height of the specimen. To check the potential for the formation of burning drops from the specimens, the filter paper was placed under the specimen, which would ignite upon contact with a burning drop from the tested specimen.

#### 2.4.3. Sound Absorption Test

**Figure 6:**
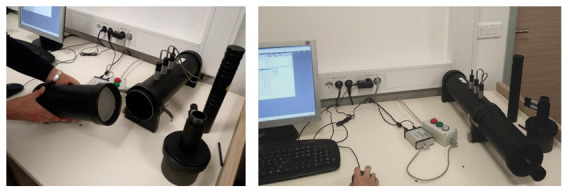
Open impedance tube with the inserted specimen (**left**) and assembled impedance tube ready to conduct the experiment (**right**).

Thermal insulation boards usually also act as a sound absorbing layer in the building element. Incident sound waves on the building element or partition are partly reflected, transmitted or absorbed. Noise reduction with the absorption of the incident sound wave is very effective and therefore it is important to define the sound absorption coefficient α of the insulation boards. In this research, the method with impedance tube, two microphones and digital frequency analyzing system was used according to the standard ISO 10534-2 [39] to determine the sound absorption coefficient for the standard specimens for normal sound incidence. An impedance tube testing instrument from the manufacturer Brüel and Kjaer (Naerum, Denmark) was used with a large tube set-up as shown in Figure 6. Therefore, the specimens with a diameter of 98.5 mm were prepared to place them in the large tube set-up. The test was conducted for the sound frequency range between 125 and 4000 Hz. The specimens for this test were prepared only from the insulation boards with a thickness of 30 mm because of the problems with the disintegration of 50 mm thick specimens when cut them into a circular shape with a diameter of 98.5 mm.

## 3. Results and Discussion

### 3.1. Thermal Conductivity Coefficient λ

**Figure 7:**
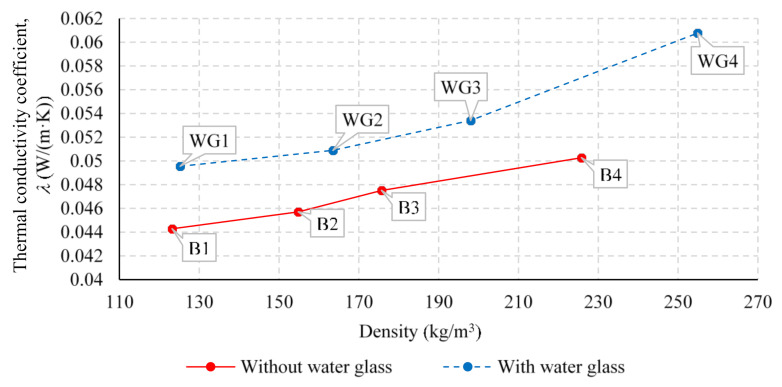
Relationship between thermal conductivity coefficient *λ* and density of the produced thermal insulation boards for specimens with a thickness of 30 mm with and without added water glass.

**Figure 8:**
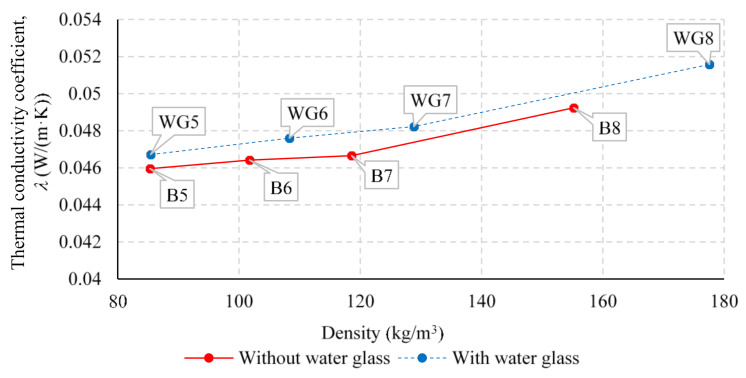
Relationship between thermal conductivity coefficient *λ* and density of the produced thermal insulation boards for specimens with a thickness of 50 mm with and without added water glass.

The relationship between the density of the produced thermal insulation boards and the thermal conductivity coefficient *λ*, for the specimens with 30 and 50 mm thickness is shown in Figure 7 and Figure 8, respectively. It is obvious that when increasing the density of the produced thermal insulation boards, the thermal conductivity coefficient *λ* increases almost linearly along with it. This is due to the lower air proportion within the specimen, which was achieved with increased density and makes the conductive heat transfer through fibers more prominent.

The thermal conductivity coefficient *λ* of the specimens without the application of water glass ranged between 0.044 and 0.050 W/(m·K), corresponding to the density range between 85.3 and 225.8 kg/m^3^. For the specimens with the application of water glass during the production of the specimen, a similar relationship can be observed, where the thermal conductivity coefficient *λ* ranges from 0.047 to 0.061 W/(m·K) for the corresponding density, which is within the range of 85.4 and 254.9 kg/m^3^. It is also clear that with the application of water glass during the production process of 30 mm thick specimens, the thermal conductivity coefficient *λ* increases by an average of 0.005 W/(m·K) compared to the specimens without added water glass. At the same time, a smaller increase in thermal conductivity coefficient *λ* can be observed when adding the water glass during the production process of 50 mm thick specimens, on average by 0.001 W/(m·K).

### 3.2. Fire Resistance Property

Within this research, special attention was paid to the reaction to fire of the produced thermal insulation. As mentioned above, two types of thermal insulation boards were produced simultaneously, where one type was treated with water glass application during production. The main reason for treating the specimens with water glass was to enhance their fire resistance and also the bonding between the shredded nonwoven waste textile fractions.

**Figure 9:**
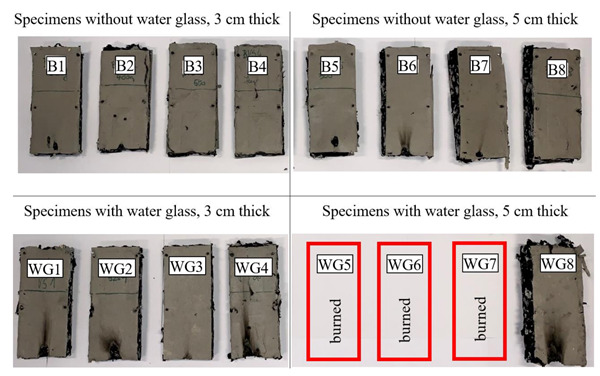
Specimens of thermal insulation boards after reaction to fire test.

The results of the reaction to fire test are shown in Table 2 and Table 3 for the 30 and 50 mm thick specimens, respectively. The specimens without water glass application tend to have shorter ignition time and show less potential for the appearance of flaming droplets in comparison to the specimen with water glass treatment. However, nonwoven polyester textile proved to be self-extinguishing when exposed to a small open flame, as the material first shrinks and then melts. This melted product consequently extinguishes the flame and the temperature drops, which prevents the appearance of flaming droplets. None of the specimens without added water glass (B1–B8) reached the flame height limit of 150 mm (Figure 9) according to the standard. However, the 50 mm thick specimens with water glass application WG5, WG6 and WG7 are missing in Figure 9 because they burned out completely and in the case of specimen WG8, the flame almost reached the control limit at the specimens’ height of 150 mm.

The flame on the thinner specimens with water glass application WG1–WG4 was stronger than on specimens B1–B4 without water glass. The results of the reaction to fire test clearly show an unfavorable behavior of the specimens with water glass application in comparison to the specimens without water glass, which is in contradiction to some other authors ([35,36,40]) who have proved that the addition of water glass can improve the fire resistance of their specific application and product. The reason for the unfavorable behavior of specimens with added water glass may be because the application of water glass to the nonwoven textile flakes and fibers locally prevents melting of the material. This preserves the structure of thermal insulation with a high proportion of air voids, and also allows the flammable smoke mixed with oxygen to flow with the chimney effect through such specimen. This is then the condition for the severe flame propagation and the complete burning out of the material, as it happened with specimens WG6, WG7, WG8.

From the experimental results, it can be concluded that in our case the treatment of the shredded polyester nonwoven textile with the addition of water glass negatively affects the fire resistance properties and therefore there is no benefit for its use.

### 3.3. Acoustic Properties

**Figure 10:**
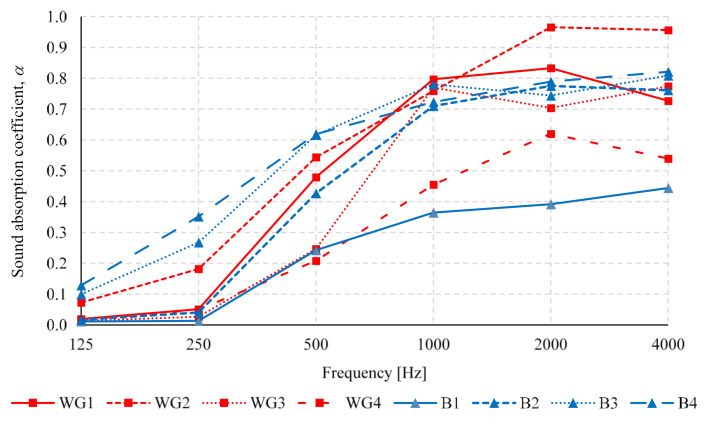
Sound absorption coefficient *α* of thermal insulation boards made of waste nonwoven textile for the octave range frequencies.

It is always beneficial for thermal insulation boards if they possess good acoustic properties. Therefore, we evaluated the sound absorption coefficient *α* for the octave range from 125 to 4000 Hz, which is a standardized evaluation range for building materials. The frequency dependent results of the sound absorption coefficient *α* for 30 mm thick specimens are shown in Figure 10.

For all tested specimens the α values at 125 Hz are below 0.15, which is an expected result for a thin fibrous material. With the specimens without water glass (B1–B4), a density dependent increase in sound absorption properties of specimens is observed. Specimen B1 with the lowest density (123 kg/m^3^) showed the worst behavior over the entire frequency range, reaching the highest sound absorption coefficient *α* = 0.44 at 4000 Hz. Specimens B1, B2 and B3 show similar absorption properties at 1000 to 4000 Hz octave bands reaching up to *α* = 0.80, and the acoustic performance of the two specimens with the highest densities (B3 and B4) is very favorable already at 250 and 500 Hz. The sound absorption quality of specimens with added water glass (WG1–WG4) similarly improves with frequency. However, the specimens with the lowest density (WG1 and WG2) show the highest *α* values, unlike the specimens without added water glass. Addition of water glass probably increases the airflow resistivity and affects the sound absorption capability of the material [41].

In addition, we evaluated noise reduction coefficient NRC for all tested specimens given in Table 4. NRC was calculated as an average sound absorption coefficient at octave bands of 250, 500, 1000 and 2000 Hz, which is a scalar representation of the sound absorption performance of the insulation boards. The overall sound absorbing performance of specimens B1, B2, B3 and B4 improves from 0.20 up to 0.60 with an increase in density of the specimens from 123.2 to 225.8 kg/m^3^. At the same time, the specimens with added water glass show a different relationship between NRC and density. Specimen WG4 with the highest density has the lowest NRC equal to 0.35. The difference in the behavior of specimens with or without water glass could be explained by the difference in airflow resistivity of two different materials. For common materials and products, NRC must be greater than 0.2 in order to classify them as sound-absorbing materials [42]. As all specimens show higher NRC than 0.2, we can conclude that all of the developed thermal insulation boards can be effectively used as sound-absorbing elements. Especially good sound absorbers are those without water glass treatment and with the highest density, or specimens with added water glass at lower densities.

### 3.4. Comparison of Developed Insulation Board with Conventional Insulation Materials

To compare the efficiency of the developed insulation materials within the present study with other conventional materials used for civil engineering applications the Table 5 was prepared. The density, thermal conductivity coefficient *λ* and noise reduction coefficient NRC are summarized and compared. The thermal conductivity coefficient *λ* of the thermal insulation presented in this study is in the range of other conventional thermal insulation materials such as rock wool mats, fiberglass mats and polystyrene boards. The NRC values of the developed insulation slabs are also similar to the conventional fibrous materials and at the same time they show favorable behavior when compared to cellular synthetic materials such as polystyrene. Other researchers ([9,11,13,15,43]) who have been dealing with the mixed waste textile and various recycling techniques have obtained similar results to the results presented in this study. There is also an option to impregnate the fibers with the aerogel which significantly reduce the thermal conductivity coefficient [33]. This type of thermal insulation is an ideal solution for the internal insulation of outer walls, where it is important to save the available area inside the rooms. However, the thermal insulation slabs made of waste nonwoven polyester textile developed in the present study proved to have adequate characteristics for the purposes of civil engineering applications.

## 4. Conclusions

The present study shows the great possibility of recycling industrial waste nonwoven polyester textile. The research was conducted in cooperation with the industrial partner FILC/Freudenberg that manufactures the nonwoven polyester textile and whose production process results in substantial amounts of waste textile. The polyester nonwoven textile was first shredded into smaller fractions that were then in a certain amount placed into a hot press and compressed to partly melt the fibers in order to bind them together into a compact thermal insulation board. Samples with the addition of water glass were prepared, which was applied with water spray in layers for the fire retardant and binding purposes. The production process proved to be very suitable for the production of high-quality compact thermal insulation boards. Basic experimental tests of the produced thermal insulation were conducted to check thermal conductivity coefficient, reaction to fire resistance and acoustic properties to justify the possibility of using the considered thermal insulation boards also as sound absorbers. Based on the experimental results, the following conclusions can be drawn:

1. Compact semi rigid thermal insulation boards can be produced with densities ranging from 123.2 to 255 kg/m^3^.
2. Thermal conduction testing proved that the specimens without water glass and with the lowest density are suitable for the purpose of providing thermal efficiency in buildings and have thermal conductivity coefficient *λ* values of 0.044 W/(m·K). The addition of water glass during the production process had a negative impact on the thermal conductivity coefficient *λ*, which increased by 0.005 up to 0.049 W/(m·K).
3. The reaction to the fire test has shown that the addition of water glass makes the product more vulnerable to open flame, which is in contradiction to the desired properties. At the same time, the specimens without water glass show a favorable self-extinguishing behavior when exposed to fire.
4. All of the produced specimens have NRC of more than 0.20 and can therefore be classified as sound absorbing products. As expected, they show low sound absorption coefficient *α* within the low frequency range. NRC increases from 0.20 to 0.60 with the increasing density of the specimen without added water glass. The specimens with the highest density proved to have the sound absorption coefficient between 0.70 and 0.80 at 1 kHz and above.

In conclusion, we have demonstrated that it is possible to produce high-quality thermal and acoustic boards from industrial waste, which is usually clean and high-quality material. The produced insulation boards are also a good example of circular economy and represent a great contribution to the concept of sustainable economy. The produced thermal and acoustic insulation can be effectively used for the insulation of outer walls, and roofs between and below the rafters and partition walls.

Instead of incineration of clean and high-quality waste polyester nonwoven textile that causes pollution and CO_2_ release, the very same material can be utilized for the thermal insulation of buildings to improve their energy efficiency, while saving additional CO_2_ release.

## Figures and Tables

**Table 1:** Composition, thickness and final density of the produced thermal insulation slab samples.

Sample	Mass of the Waste (g)	Mass of the Water Glass (g)	Designed Thickness (cm)	Density (kg/m^3^)
B1	300	0	3	123
B2	400	0	3	155
B3	500	0	3	176
B4	700	0	3	226
B5	300	0	5	85
B6	400	0	5	102
B7	500	0	5	119
B8	700	0	5	155
WG1	300	100	3	125
WG2	400	125	3	164
WG3	500	150	3	198
WG4	700	200	3	255
WG5	300	100	5	85
WG6	400	125	5	108
WG7	500	150	5	129
WG8	700	200	5	178

**Table 2:** Resistance to fire properties for the specimens with a thickness of 30 mm.

Specimen	Ignition Time (s)	Flaming Droplet Time (s)	Flame Reaching the Reference Line Time (s)	Fire Extinguishing Time (s)
B1	2	/	/	40
B2	/	/	/	/
B3	15	/	/	32
B4	/	/	/	/
WG1	3	22	/	27
WG2	10	55	/	60
WG3	15	/	/	30
WG4	13	30	/	69

**Table 3:** Resistance to fire properties for the specimens with a thickness of 50 mm.

Specimen	Ignition Time (s)	Flaming Droplet Time (s)	Flame Reaching the Reference Line Time (s)	Fire Extinguishing Time (s)
B5	3	10	/	25
B6	5	/	/	28
B7	14	22	/	24
B8	/	/	/	/
WG5	5	10	90	/
WG6	7	13	120	/
WG7	5	15	130	/
WG8	13	24	/	132

**Table 4:** Noise reduction coefficient NRC for the insulation boards with and without water glass application with a thickness of 30 mm.

Specimen	Density (kg/m^3^)	NRC
B1	123.2	0.25
B2	154.8	0.50
B3	175.7	0.66
B4	225.8	0.66
WG1	125.2	0.55
WG2	163.5	0.60
WG3	198	0.45
WG4	254.9	0.35

**Table 5:** Summary of density, thermal insulation coefficient *λ* and noise reduction coefficient NRC for the developed insulation boards and various conventional insulation materials.

Specimen	Density (kg/m^3^)	Thermal Conductivity Coefficient, λ (W/(m·K))	NRC	Source
B1–B8	85–226	0.044–0.05	0.25–0.66	This study
WG1–WG8	85–255	0.047–0.061	0.35–0.55	
Mixed textile waste	50–500	0.034–0.08	43–80	[9,11,13,15,43]
Polyester fibers impregnated with aerogel	50–70	0.0255–0.0281	/	[32]
Rockwool mat	30–180	0.038–0.039	0.65	[44,45]
Fiberglass mat	14–80	0.032–0.038	0.65	[44,45]
Extruded polystyrene	33	0.035–0.038	/	[45]
Polystyrene	15–30	0.041	0.17	[42,45]
Polyurethane foam	15–80	0.025–0.040	/	[45,46]

## Data Availability

All data appear in the submitted article.
